# Polyhydroxyalkanoates (PHA) genes Database

**DOI:** 10.6026/97320630015036

**Published:** 2019-02-03

**Authors:** Mohammad Alamgeer

**Affiliations:** 1 Department of Information Systems, King Khalid University, Kingdom of Saudi Arabia (KSA)

**Keywords:** Polyhydroxyalkanoates, Database, Gensolution, PHA, Plastics, Biosynthesis, Biodegradable Plastics, Biodegradable Polymers, CAB Genes

## Abstract

Polyhydroxyalkanoates (PHA) are polyesters of various hydroxyl alkanoates that are synthesized by many gram-positive and gramnegative bacteria from about 75 different genera. PHA genes database is a repository of genes and its genomic information related to PHA.It contains data on the genomic characterization of intermediates of PHA. These include CAB genes, responsible for biodegradable plastic synthesis. The genomic database provides data on PHA genes from archaeal, bacterial and eukaryotic genomes.

## Background

Plastics are the most widely used synthetic polymers [1]. Due to their non-degradative nature, synthetic polymers have become an environmental eyesore. Biodegradable plastics like Polyhydroxyalkanoates (PHAs) comprise a group of natural biodegradable polyesters that are synthesized by microorganisms. PHAs were discovered in prokaryotes as carbon and energy storage materials [2]. Plastics being xenobiotic are recalcitrant to microbial degradation [3]. They have gained major importance due to their structural diversity and close analogy to plastics [4]. They have promising properties such as high biodegradability in different environments. Many microorganisms using intracellular or extracellular PHA depolymerases can degrade PHA. PHA depolymerases are very diverse in sequence and substrate specificity but share a common a/β-hydrolase fold and a catalytic triad, which is also found in other a/β-hydrolases [5]. PHA, a biodegradable plastic, was produced in microorganisms and was first discovered by Lemoigne in 1925. It has a relatively high melting point and it gets crystallized rapidly. It has high melting temperature (175°C) and relatively high tensile strength (30-35 MPa) [6].

The PHB biosynthetic genes phbA (for 3-ketothiolase), phbB (NADPH-dependent acetoacetyl-CoA reductase) and phbC (PHB synthase) are clustered and organized in one phbCAB operon [4] but the similarity in the mechanisms of regulation of these divergent operons is yet unknown. Structural studies will further improve our understanding of the mechanism of action of these enzymes and aid us in improving and selecting better candidates for increased production. Study on the enzyme PHA synthase, activity of extremely halophilic archaeon, Haloarculamarismortui, has suggested that PHA is constitutively expressed independent of nutrient rich or nutrient-limited conditions [7]. PHAs are produced in organisms under certain conditions with the help of the following enzymes: i) β-ketoacyl CoA thiolase (PhaA - EC 2.3.1.9), ii) NADPH dependent Acetoacetyl CoA reductase (PhaB - EC 1.1.1.36) and iii) PHA synthase (PhaC - EC 2.3.1.41). PHAs are gaining attention among biodegradable polymers due to their promising properties such as high biodegradability in different environments. 253 sequenced genomes have been used for phylogenetic and statistical analyses of 3 genes, which are involved in PHA biosynthesis. There are about 24 organisms with an ability to acquire and adapt these genes from taxonomically distant relatives primarily through horizontal gene transfer (HGT) events. Microbes acquire or lose genetic material in an effort to encounter adverse environmental conditions. They undergo modification(s) of the existing regulatory mechanisms(s) or develop novel operons. These organisms may prove more amenable to genetic modifications using recombinant DNA technology. These organisms have the ability to use a wide range of industrial wastewater to degrade environmental pollutants. Thus they are use to both breakdown wastes and produce PHAs. The PHA genes database supports query search on PHA biosynthetic genes by maintaining a comprehensive, nonredundant, well organized and freely available in a relational database. The entries in the database are clustered into different taxa.

## Features of the PHA genes Database:

We created the database to facilitate compositional analysis and provide additional evidence for discussing the possible origin of PHA genes. The current version of the database contains organisms that are sorted alphabetically and classified taxonomically. It is the first database on genes of PHA metabolism extracted from the sequenced genomes. This database is helpful for research on biodegradable plastics. It provides information on G+C content, codon usage (Relative Synonymous Codon Usage, RSCU), χ^2^ (Chi- Square) values for each gene and its host genome for ready use.

Two major independent modules are designed which are useful to analyze the CAB genes of organism from the database. The database web page is designed which is user friendly and completely validated. The home page of PHA genes database provides facilities to query search by entering search keywords of organism's name or by clicking on alphabetic list of organisms /taxons.

The PHA database has the following features:

## 1.Non-redundancy:

The database is non-redundant. The redundancy is fully controlled for improving the performance of queries and for saving storage space on the database server.

## 2.Classification:

The entries in the database are grouped in 23 different taxons. The organisms having phaCAB gene(s) are linked with taxon from which it belongs taxonomically. The records of the gene in organism sub-group are identified by unique record ID. Each record ID has associative amino acid record as well as preceding and succeeding gene record with the respective sequence.

## 3.Cross references:

PHA records are cross-referenced to NCBI database [8]. The enzymes located on reference pathway of Butanoate metabolism are also cross-referenced to the KEGG [9] and Biocarta [10] databases.

## 4.Information retrieval:

The database serves as a major information resource to support biodegradable investigations in the area of plastic synthesis. Retrieval and knowledge discovery are facilitated by search options (prompting organism name, present alphabetic list of the organism and hierarchy of taxons). The alphabetic list makes it possible to rapidly retrieve information on organism of pha genes.

## 5. Architecture model:

The PHA genes database is based on the Client/Server 3-Tier architecture-computing model. The TP Monitor is used as a middle layer to establish the link between Client's request and Server's response.

## Database Content and Access:

The PHA genes database is created in order to store all the sequence and statistical parameters of those genes, which are involved in the PHA metabolism pathway for bioplastics synthesis in a single place. This database also includes the preceding and succeeding genes of phaCAB genes present on genome map. The database also provides storage place of organism and taxon in which the genes are clustered.

Currently, there are 23 taxons, 233 organisms and 381 (phaA -111, phaB -199, phaC - 71) genes entries in the database. Each gene has nucleotide sequence, amino acid sequence and the details of preceding and succeeding genes with associated parameters. The PHA genes database home page at the GenSolution website provides space to prompt the organism name. For easy access, home page provides alphabetic list of organism names. The taxon link displays the hierarchy list of taxons and nested organisms. The user will get the result after entering the organism name or on clicking resultant organism name. The result page provides phaCAB (phaA, phaB, phaC) genes in separate column with parameters in rows. The search results also provide links to the nucleotide and amino acid sequence of individual genes and details of preceding and succeeding genes. The GI Number (located amino acid, preceding and succeeding gene result page) links with NCBI database for associated gene. Pathway Information hyperlink provides the reference pathway of Butanoate metabolism for PHA production.

## Database Organization:

The PHA genes database which is available at the GenSolution website is non-redundant. All the operations on the query search are basically performed using the organism name. The genotype(s) of the respective organism ID (maintained by the database organization) are found from specific search module. It gives the detail information of the gene(s) - phaCAB. The cross-reference for individual enzymes of a particular phaCAB gene with NCBI database provides more details for gene and sequence. Each result page provides hyperlinked id for respective amino acid sequence as well as the preceding and succeeding gene. All the organism records are maintained by super-group of referenced taxon. Data integrity and hyperlinked reference ID are run on all entries standardization rules are revised; such type of cross check is maintained by using reserved id of each entities.

Standardization rules and controlled vocabularies are applied for organism names, gene names, genetic information and all other fields. The used keywords to represent all information of the database are derived from many sources where possible. The GenSolution website provides the details of the used keywords.

## Data Extraction:

The list of taxon and organisms name has been downloaded NCBI and KEGG genome databases. Two global databases (KEGG and Biocarta) have a collection of metabolic pathways, which helps to identify the CAB genes responsible for PHA. The NCBI database helps to collect properties of all genes, which are present in PHA database.

## Availability:

The PolyHydroxyAlkanoates genes Database is freely available on GenSolution website which is accessible at http://www.gensolution.org/pha_gene_db.asp or http://gensolution.az-group.org/pha_gene_db.asp. All comments, queries and correction should be sent by email to gensolution@azgroup. org or dralamgeer@az-group.org. The website provides feedback and contacts link on home page of GenSolution website (http://www.gensolution.org).
